# In Vivo Evaluation of Mechanically Processed Stromal Vascular Fraction in a Chamber Vascularized by an Arteriovenous Shunt

**DOI:** 10.3390/pharmaceutics14020417

**Published:** 2022-02-14

**Authors:** Bong-Sung Kim, Shih-Heng Chen, Mauro Vasella, Marco Guidi, Epameinondas Gousopoulos, Nicole Lindenblatt, Huang-Kai Kao

**Affiliations:** 1 Division of Reconstructive Microsurgery, Department of Plastic and Reconstructive Surgery, Chang Gung Memorial Hospital, College of Medicine, Chang Gung University, Taoyuan City 333, Taiwan; shihheng@mac.com; 2 Department of Plastic Surgery and Hand Surgery, University Hospital Zurich, 8091 Zurich, Switzerland; mauro.vasella@usz.ch (M.V.); marco.guidi@usz.ch (M.G.); epameinondas.gousopoulos@usz.ch (E.G.); nicole.lindenblatt@usz.ch (N.L.)

**Keywords:** stromal vascular fraction, adipose-derived stem cells, adipose-derived stromal cells, mechanical isolation, tissue engineering, arteriovenous loop, arteriovenous shunt

## Abstract

Mechanically processed stromal vascular fraction (mSVF) is a promising source for regenerative purposes. To study the in vivo fate of the mSVF, we herein used a vascularized tissue engineering chamber that insulates the target mSVF from the surrounding environment. In contrast to previous models, we propose an arteriovenous (AV) shunt between saphenous vessels in rats without a venous graft. Mechanical SVF was processed from the fat pads of male Sprague Dawley rats, mixed with a fibrin hydrogel and implanted into an inguinal tissue engineering chamber. An arteriovenous shunt was established between saphenous artery and vein. On the contralateral side, an mSVF-fibrin hydrogel mix without vascular axis served as a non-vascularized control. After two and six weeks, rats were sacrificed for further analysis. Mechanical SVF showed significant numbers of mesenchymal stromal cells. Vascularized mSVF explants gained weight over time. Perilipin and CD31 expression were significantly higher in the mSVF explants after six weeks while no difference in DAPI positive cells, collagen deposition and FABP4 expression was observed. Morphologically, no differentiated adipocytes but a dense cell-rich tissue with perilipin-positive cells was found after six weeks. The phosphorylation of ERK1/2 was significantly enhanced after six weeks while Akt activation remained unaltered. Finally, mSVF explants stably expressed and released VEGF, bFGF and TGFb. Vascularized mSVF is able to proliferate and express adipocyte-specific markers. The AV shunt model is a valuable refinement of currently existing AV loop models in the rat which contributes to the fundamental 3R principles of animal research.

## 1. Introduction

Adipose tissue has advanced to one of the major tools in regenerative medicine including regenerative plastic surgery and tissue engineering. The discovery of adipose-derived stem cells, or rather, adipose-derived stromal cells (ASCs) [1], has had a particularly incomparable impact as ASCs mediate effects comparable to those of other mesenchymal stem cells (MSCs), though with a much more acceptable donor site morbidity. ASCs have been examined for uncountable clinical applications such as wound healing, tissue augmentation, skin rejuvenation, neurological and gastrointestinal disease, cardiovascular disease, musculoskeletal diseases and many more [2,3]. Additionally, ASCs appear to be a promising cell source for tissue engineering, e.g., by cultivating ASCs on scaffolds to replace, modify or augment desired tissue [4]. Despite the excitement generated over the years, methodological and regulatory issues have significantly hampered the clinical translation of ASCs. The required enzymatic dissociation to isolate the stromal vascular fraction (SVF) and subsequent cultivation resulting in the final ASC population are time-consuming, expensive and demanding with regard to infrastructure. Beyond that, in most Western countries, collagenase digestion and cultivation are classified as cell manipulations that underlie strict regulation, e.g., by the US Food and Drug Administration (FDA) or European Medicines Agency (EMA) [5].

With the goal to circumvent the aforementioned regulatory issues and provide a more accessible progenitor cell isolation from adipose tissue, several authors have proposed mechanical SVF (mSVF) isolation techniques. These methods circumvent regulatory restraints by utilizing mechanical forces without the employment of enzymes and skipping the lengthy cultivation process [6]. Several mechanical protocols were suggested which include shaking [7], extended centrifugation [8], vibration [9], cutting with blades [10], emulsification [11] and many more. In 2019, the International Federation for Adipose Therapeutics and Science (IFATS) stressed the particular importance of mechanically processed SVF [12].

We have recently introduced an mSVF isolation protocol (also called the “lipoconcentrate” protocol) as a modification of the much propagated and most widely used mechanical isolation protocol named the “nano-fat” technique by Tonnard et al. that suggests SVF isolation by shifting fat grafts between syringes through a connector with a small diameter, disrupting the relatively large adipocytes [11]. In our approach, the nano-fat is additionally centrifuged resulting in an adipocyte-depleted, ASC-rich mSVF product [13]. The mSVF can be directly injected to induce soft tissue rejuvenation and scar remodeling or used for tissue engineering purposes [14]. Although basic parameters of our mSVF protocol including SVF cell count, surface marker expression and release of growth factors/enzymes have been determined in vitro, the fate of the mSVF requires additional in vivo evaluation.

In the present study, we aimed to expand the already collected in vitro data [13] on the feasibility of our mechanical SVF isolation protocol in an in vivo approach. It was our goal to understand the biological behavior and fate of mSVF once it is implanted in vivo over a prolonged period of time. The simplest methods to investigate mSVF cells in an in vivo setting are a subcutaneous injection of mSVF into rodents, as comprehensively reviewed by Lujan-Hernandez et al. [15]. However, a subcutaneous injection without advanced cell labeling also renders it difficult to distinguish the transplanted mSVF cells from the surrounding recipient tissue at the time of explantation. To specifically observe the implanted mSVF cells and insulate the mSVF from its surrounding tissue at the recipient site, we inserted the mSVF into a closed artificial tissue engineering chamber. By placing an arteriovenous (AV) shunt (AVS), the mSVF within the chamber was vascularized. This proposed technique was adapted from established tissue engineering models [16,17,18,19,20]. However, in contrast to existing models, we propose an AVS of the saphenous vessels without a graft that is usually taken from the contralateral leg. We hypothesized that mSVF, once it is implanted into our vascularized model in rats, will survive over the period of six weeks and slowly grow to generate de novo adipose tissue. We further postulated that the AVS model without a graft is a reliable model that contributes to the 3R principles of animal experimentation [21].

## 2. Materials and Methods

### 2.1. Rats

All in vivo experiments were performed by the same author (B.-S.K.) and were approved by the Chang Gung Memorial Hospital Linkou institutional review board (approval number IACUC2018122018, approval date 4 November 2020). A total of 13 male Sprague Dawley rats with an average weight of 380–420 g and an age of 12–14 weeks were purchased from the National Laboratory Animal Center in Taipei, Taiwan and housed at the animal facility of the Chang Gung Memorial Hospital Linkou.

### 2.2. Fat Harvest and Processing

**Figure 1:**
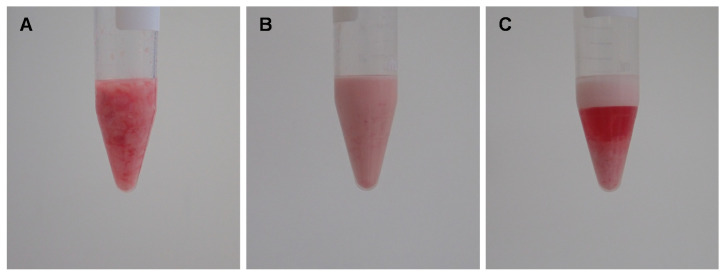
Creating mSVF by a combination of emulsification and centrifugation. Adipose tissue of male Sprague Dawley rats was harvested from both inguinal fat pads. (**A**) After removing connective tissue and blood vessels, the fat was finely minced. (**B**) Minced fat was shifted thirty times through a 1.2 mm tunnel to emulsify fat. (**C**) Emulsified fat was centrifuged, the mSVF is the bottom layer.

Rats were anesthetized by 5% isoflurane for induction. The rats were placed on their backs. Both inguinal areas and legs were clipped and disinfected. Bilateral inguinal fat pads were en bloc harvested and immediately processed in the laboratory. The fat was processed according to our earlier published protocol [13]. Blood vessels and visible connective tissue of the en bloc inguinal fat pads were cautiously removed, and fat was rinsed with sterile 0.9% saline solution. Next, the fat was finely minced by a scalpel to imitate the fragmentation that fat undergoes during the liposuction procedure in humans. The minced fat (Figure 1A) was then shifted through a Luer lock connector with an inner diameter of 1.2 mm (Tulip Medical Products, San Diego, CA, USA) for thirty times, resulting in emulsified fat (Figure 1B). The emulsified fat was finally centrifuged at 1200× *g* for 5 min which resulted in the final mSVF found at the bottom of the centrifugation tube (Figure 1C). The upper layers were discarded.

### 2.3. AV Shunt and Implantation of mSVF-Fibrin Hydrogel Mix

**Figure 2:**
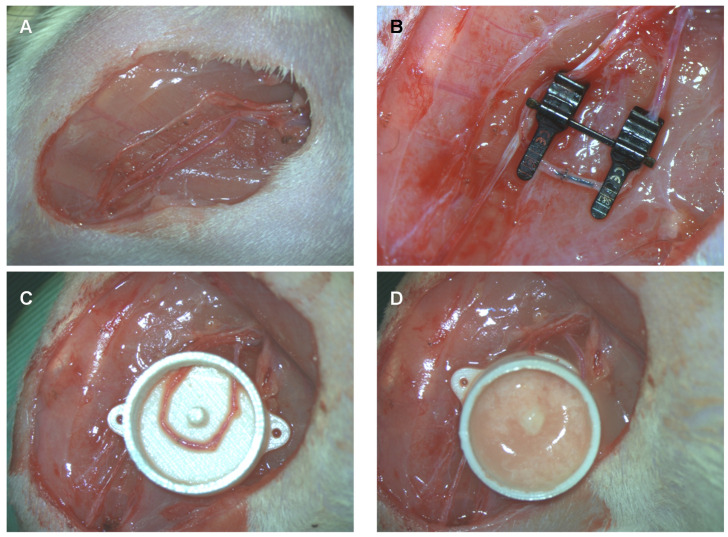
Arteriovenous shunt of the saphenous artery and vein in rats. (**A**) The saphenous artery and vein as well as the obturator nerve were dissected on each leg. (**B**) On the experimental side, the saphenous artery and vein are anastomosed in the knee region just proximally to their bifurcation. On the control side, no AV shunt is established. To prevent stitching of the back wall, a black 4/0 nylon suture is inserted into the lumen and removed before tying the last stitch. (**C**) The AV shunt is placed into a polycarbonate tissue engineering chamber. (**D**) The chamber is filled with an mSVF-fibrin hydrogel mix and closed by a lit. On the control site, the chamber is filled with an mSVF-fibrin hydrogel mix which is not vascularized by an AV shunt.

While the fat was processed, AV shunting was performed in parallel. All surgeries were performed under a surgical microscope under sterile conditions on a heat mat. For the experimental group, an AV shunt based on the saphenous vessels was established. The saphenous artery, saphenous vein and obturator nerve were dissected from their origin down to the knee under ligation of all branches (Figure 2A). The saphenous artery and vein were ligated just proximal to their distal bifurcation. The artery and vein were flushed with heparin solution. A double clamp was applied, and an AV shunt was created by an end-to-end anastomosis between the distal ends of the artery and vein with 11/0 nylon. To avoid back-wall stitches, a short 4/0 nylon suture was temporarily inserted into the lumen (Figure 2B). The double clamp was released, and patency was checked. The obturator nerve was preserved.

Next, a polycarbonate tissue engineering chamber (total volume of 0.9 mL with a central pin to secure the AV shunt) was placed through a small entrance at one side (Figure 2C). An mSVF-fibrin hydrogel mix was filled into the chamber. To create the mSVF-fibrin hydrogel mix, 0.3 mL of autologous mSVF was mixed with 0.3 mL of fibrin hydrogel (10 IU thrombin (Sigma-Aldrich, Burlington, VT, USA), 12.5 mg/mL fibrinogen (Sigma-Aldrich, Burlington, VT, USA)). Half of the solidified mSVF-fibron hydrogel mix was injected beneath and the other half above the AV shunt (Figure 2D). The chamber was closed by a lit and secured to the thigh with two 4/0 nylon sutures. Skin closure was completed by interrupted Vicryl 4/0 sutures.

For the contralateral side, the saphenous artery, vein and nerve (on the contralateral leg to the experimental group) were dissected but no AV shunting was performed. Instead, a same-sized tissue engineering chamber was placed into the thigh and filled with the equal amount of the autologous mSVF-fibrin hydrogel mix. The skin was closed in an identical fashion.

In case a thrombosis of the AV shunt was seen during the surgery, another AV shunt was performed on the contralateral side and the failed AV shunt was used as a control.

Chambers were collected after two weeks and six weeks for further analysis.

### 2.4. Histology, Immunohistochemistry and Immunofluorescence

For histology, immunohistochemistry (CD31) and immunofluorescence (perilipin), established protocols were used [22]. In short, 3 µm-thick sections were deparaffinized and rehydrated followed by microwaving with sodium citrate. Paraffin-embedded and formalin-fixed 3 µm sections were stained by hematoxylin and eosin (HE) and Masson’s trichrome.

For immunohistochemistry and immunofluorescence, unspecific antigens were blocked by bovine serum albumin (BSA). For immunohistochemistry, sections were next incubated with primary CD31 (Abcam, Cambridge, UK) and secondary antibodies (HRP, Dako, Agilent Technologies, Santa Clara, CA, USA) followed by HE counterstaining. For immunofluorescence, a primary antibody against perilipin (Cell Signaling Technology, Danvers, MA, USA) was used. Finally, the sections were incubated with fluorescent AF488/AF594 secondary antibodies CD31 (Abcam, Cambridge, UK) and counterstained with diamidino-2-phenylindole (DAPI, Sigma-Aldrich, Burlington, VT, USA).

### 2.5. Flow Cytometry

In flow cytometry, freshly harvested fat was immediately processed to acquire mSVF. The mSVF cells were stained for the hematopoietic surface marker CD45-FITC (BD Biosciences, Franklin Lake, NJ, USA), the mesenchymal stromal cell marker CD34-PE-Cy7 (Santa Cruz Biotechnology, Inc., Dallas, TX, USA) and the endothelial marker CD31-AF647 (BD Biosciences, Franklin Lake, NJ, USA). Flow cytometric analysis was performed on a BD LSRFortessa^TM^ (BD Biosciences, Franklin Lakes, NJ, USA) flow cytometer.

### 2.6. Western Blot

Explanted mSVF was homogenized by sonication and lysates were acquired by lithium dodecyl sulfate (LDS)/ dithiothreitol (DTT) buffer LDS/DTT buffer (25% (*v*/*v*) NuPAGE lithium LDS sample buffer (Invitrogen, Karlsruhe, Germany), 50 mM DTT (Sigma-Aldrich, Munich, Germany), bidistilled water (ddH_2_O)). Sodium dodecyl sulfate-polyacrylamide gel electrophoresis (SDS-PAGE) for Akt (55 kDa), pAKT (56–60 kDa), ERK/pERK-1/2 (42–44 kDa), Actin (43 kDa) and α-tubulin were performed on 10% polyacrylamide gels. For Western blotting, the primary antibodies Akt (Abcam, Cambridge, UK), pAkt (Abcam, Cambridge, UK), ERK1/2 (Cell Signaling Technology, Danvers, MA, USA), pERK1/2 (Cell Signaling Technology, Danvers, MA, USA), actin (Merck& Co., Kenilworth, NJ, USA), α tubulin (Abcam, Cambridge, UK) and the secondary antibodies HRP-goat anti-mouse IgG, HRP-goat anti-rabbit IgG and HRP-rabbit anti-rat IgG from Abcam, Cambridge, UK were used. HRP on the blots was detected by ECL substrate (Bio-Rad Laboratories, Hercules, CA, USA).

### 2.7. Elisa

Fresh mSVF pellet was suspended in minimum essential medium (MEM; Thermo Fisher Scientific, Waltham, MA, USA) supplemented with 10% fetal bovine serum (FBS; Thermo Fisher Scientific, Waltham, MA, USA) and 1% penicillin-streptomycin. Conditioned medium was collected from SVF cells and after two days of single culture without media change and was analyzed immediately by following ELISA kits: basic fibroblast growth factor (bFGF; RayBiotech, Peachtree Corners, GA, USA; #ELR-bFGF-1; detection range 2–500 pg/mL), vascular endothelial growth factor A (VEGF-A; RayBiotech; Peachtree Corners, GA, USA; #ELR-vEGF-1; detection range 2–200 pg/mL) and tumor growth factor beta (TGF-b; Invitrogen, Carlsbad, CA, USA; TGF beta-1 Rat ELISA Kit; detection range: 31.25–2000 pg/mL).

### 2.8. Quantitative PCR (qPCR)

Quantitative PCR was performed according to published protocols [22]. Mechanical SVF explants were homogenized and mRNA was isolated by the RNeasy^®^ Mini Kit (Qiagen, Valencia, CA, USA) followed by reverse transcription with the SuperScript^®^ III First-Strand Synthesis System (Invitrogen, Carlsbad, CA, USA). Quantitative PCR was performed by the TaqMan Gene Expression Assay (Applied Biosystems, Foster City, CA, USA) on an ABI Prism^®^ 7300 system (Applied Biosystems, Foster City, CA, USA). The mRNA expression of fatty acid-binding protein 4 (FABP4; also known as adipocytes protein 2 (aP2); Rn00670361_m1), bFGF (Rn00570809_m1), TGFb (Rn00572010_m1), VEGF-A (Rn01511602_m1) and Collagen 1α1 (Rn01463848_m1) was standardized to the two housekeeping genes beta actin (Rn00667869_m1) and 18S (Rn03928990_g1) [23]. All primers were purchased from Applied Biosystems (Foster City, CA, USA).

### 2.9. Statistics

Histological images were assessed by Image J (National Institutes of Health, Bethesda, MD, USA). Comparisons between two groups were performed by the Mann–Whitney U test. Comparisons between more than two groups were performed by the Kruskal–Wallis test with Dunn’s posttest. All analysis was performed by GraphPad Prism (San Diego, CA, USA). A *p* value < 0.05 was considered as significant.

## 3. Results

### 3.1. Operated Rats

A total of six rats were operated on in the two-week group and another six rats in the six-week group. One rat of the six-week group had to be sacrificed earlier as the chambers were bilaterally exposed and the AV shunt was thrombosed. One non-vascularized chamber was lost in one of the rats that was sacrificed after two weeks.

### 3.2. Basic Characterization of mSVF by Flow Cytometry and HE Staining

**Figure 3:**
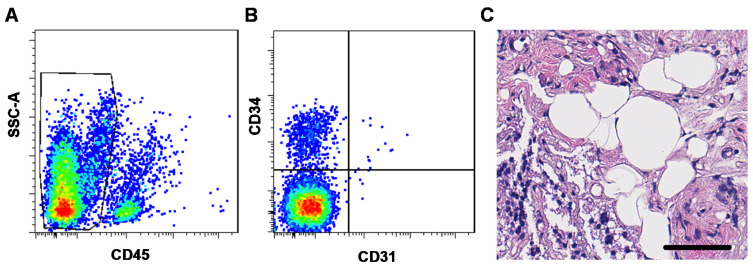
(**A**,**B**) Flow cytometry and (**C**) HE staining of mSVF cells. (**A**) and (**B**) Freshly isolated and processed mSVF cells underwent flow cytometry staining for the hematopoietic marker CD45, the endothelial marker CD31 and the mesenchymal stromal cell marker CD34. ASCs were defined as (**A**) CD45-, (**B**) CD31- and CD34+ cells and constituted an average of 7.8 ± 2.2% of all cells in the mSVF. (**C**) HE stains of freshly isolated mSVF cells mixed with fibrin hydrogel were evaluated. The mSVF showed disruption of adipose tissue with SVF cells dispersed in the fibrin hydrogel but also fragments of intact adipocytes. Bar: 50 µm.

Non-implanted mSVF-hydrogel mixes were investigated by flow cytometry and HE staining to evaluate the ASC content and histological features of the mSVF. Flow cytometry analysis revealed that around 10% of the mSVF cells were ASCs, which were defined as cells which were negative for the hematopoietic marker CD45 and the endothelial marker CD31 but positive for the mesenchymal stem cell marker CD34 (Figure 3A,B). A total of four samples were evaluated and showed an average of 7.8 ± 2.2% of CD45-, CD31-, CD34+ ASCs.

In the HE staining (Figure 3C), mSVF-hydrogel mixtures showed a clear disruption of the usual adipose tissue structure with small fragments with intact adipocytes indicating that the shifting maneuver does not lead to a complete disruption of adipocytes as suggested in the initial nano-fat protocol by Tonnard et al. in rat adipose tissue [11].

### 3.3. The Effect on Tissue Vascularization on Weight and Tissue Structure

**Figure 4:**
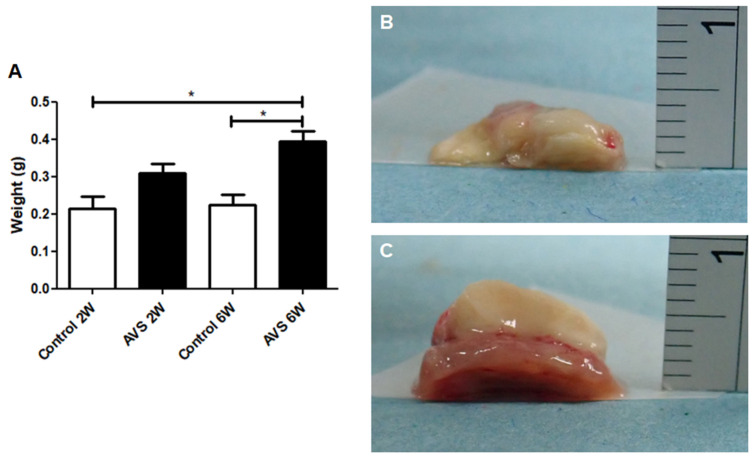
Weight gain after two and six weeks. The weight of vascularized and non-vascularized mSVF explants (without the chamber) was measured immediately after explantation and (**A**) show a significant increase in weight in the vascularized mSVF explants after six weeks as well as a significant weight difference between vascularized and non-vascularized mSVF explants after two and six weeks. Representative photographs of (**B**) non-vascularized mSVF explants after six weeks with macroscopic signs of necrosis and (**C**) vascularized mSVF explants after six weeks with viable tissue and only marginal necrosis on the top of the explants are shown. * *p* < 0.05.

The weight of the mSVF was measured after two and six weeks in vascularized and non-vascularized mSVF explants (statistical analysis in Figure 4A). A significant difference between the weight of mSVF explants with no vascularization (Figure 4B) and mSVF that were vascularized by an AV shunt (Figure 4C) was observed after six weeks. Importantly, the weight difference between vascularized mSVF explants after six weeks was markedly higher (but did not reach statistical significance; *p* = 0.1173) than vascularized mSVF explants after two weeks, whereas no difference was seen in the weight of non-vascularized mSVF explants between two and six weeks. After six weeks, the vascularized explants were significantly higher than the non-vascularized explants. Macroscopically, the non-vascularized mSVF explants were necrotic, whereas only marginal necrosis was found in vascularized mSVF explants (Figure 4).

**Figure 5:**
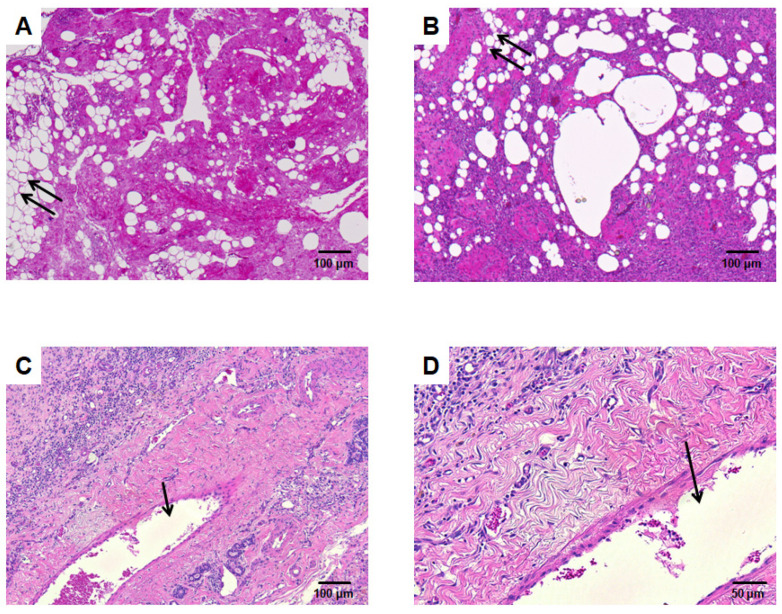
HE staining. (**A**) Mechanical SVF without AV loop explanted after 6 weeks. There are several parts that resemble adipocytes with large vacuoles (double arrow). (**B**) Mechanical SVF with AV shunt explanted after two weeks, while the adipocytes-like vacuoles decrease the interstitium is infiltrated by cells. (**C**) (lower magnification) and (**D**) (higher magnification) depict mechanical SVF supplied by an AV shunt after 6 weeks. A dense cell-rich tissue with no vacuoles is found. The arrow marks the tangentially cut AV shunt lumen.

Furthermore, in the vascularized mSVF explants, a dense cell-rich tissue was seen after two and six weeks (Figure 5). Interestingly, no mature adipocytes with a typical vacuole and peripheral nucleus were found in the vascularized mSVF explants after neither two nor six weeks. In the non-vascularized mSVF explants, by contrast, fat necrosis with cell-deployed interstitial tissue was found.

### 3.4. Collagen Deposition

**Figure 6:**
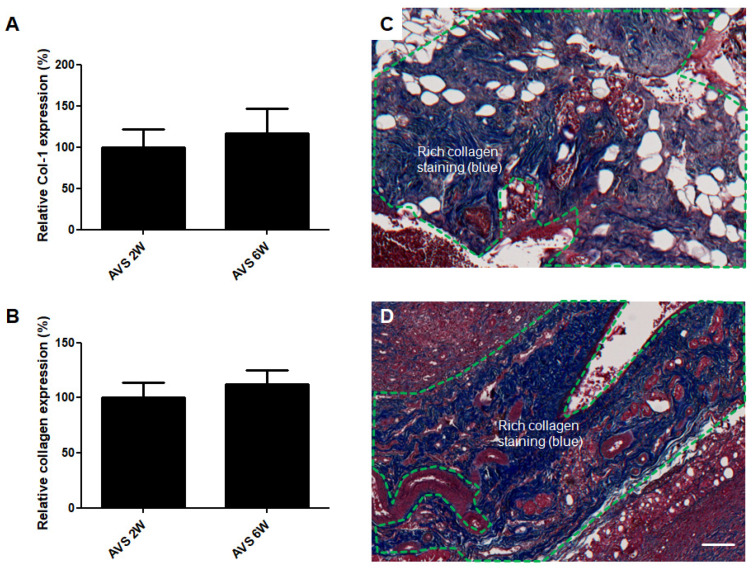
Collagen deposition of AVS-vascularized mSVF explants after two and six weeks. (**A**) No difference in collagen 1α1 mRNA levels was observed in mSVF explants after two and six weeks. (**B**) Next, trichrome staining was performed to visualize collagen expression in AVS-vascularized mSVF explants after two and six weeks. Statistical analysis revealed no difference in collagen deposition. Representative trichrome stainings of vascularized mSVF after (**C**) 2 weeks and (**D**) 6 weeks are depicted. The green dotted line demonstrates similar blue collagen staining after two and six weeks. Bar: 100 µm.

Next, Masson’s trichrome staining was utilized to evaluate the collagen matrix deposition in the vascularized mSVF explants after two and six weeks. The density of collagen was comparable between the mSVF explants at week two and six (Figure 6). After two and six weeks, the collagen was ubiquitously found in the mSVF explants with cells scattered in the extracellular matrix (ECM). The mRNA expression of collagen 1A1 was also studied by qPCR and showed no significant difference between vascularized mSVF explants at week two and six (Figure 6).

### 3.5. CD31 Expression

**Figure 7:**
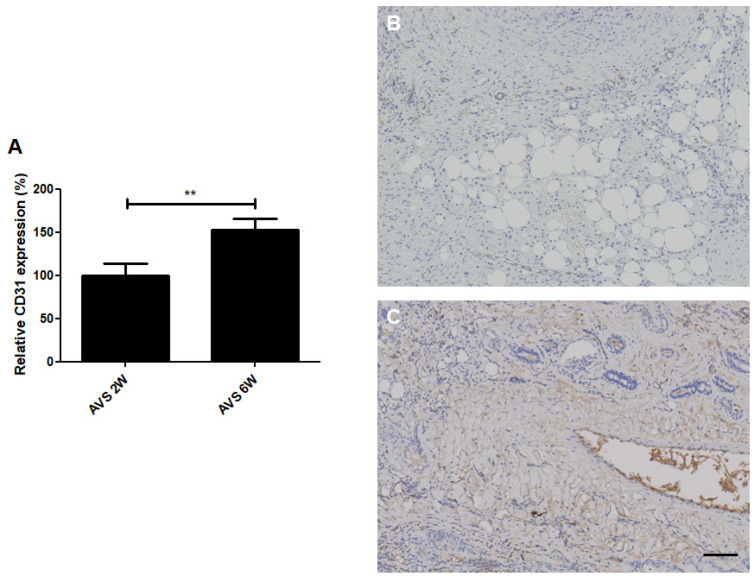
CD31 (angiogenesis) staining of vascularized mSVF explants after two and six weeks. (**A**) Statistical analysis of CD31 staining comparing vascularized mSVF explants after two and six weeks. Representative CD31 stainings of vascularized mSVF after (**B**) 2 weeks and (**C**) 6 weeks are depicted with brown color indicating positive CD31 expression. Bar: 100 µm. ** *p* < 0.01.

Immunohistochemical CD31 staining was performed to examine the expression of the endothelial marker CD31 in the vascularized mSVF explants after two and six weeks (Figure 7). The staining showed a significant increase in CD31 expression in the mSVF groups that were harvested after six weeks. Again, CD31 expression was found throughout the explants.

### 3.6. DAPI Staining and Perilipin Expression

**Figure 8:**
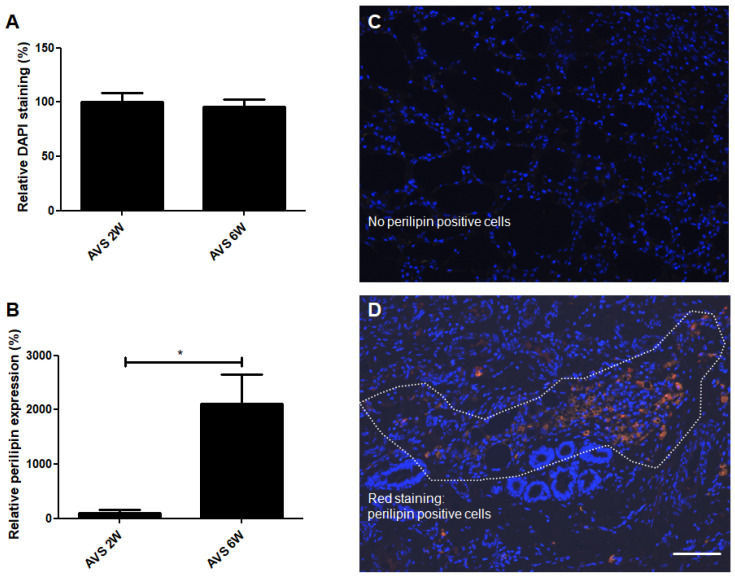
DAPI (cell nuclei) and perilipin (adipocytes) co-staining of vascularized mSVF explants after two and six weeks. (**A**) Statistical analysis of DAPI staining of vascularized mSVF explants after two and six weeks showed no statistical difference. (**B**) A statistical evaluation of perilipin staining, by contrast, demonstrated a significant increase in perilipin expression after six weeks. Representative DAPI (blue) and perilipin (red) co-staining of mSVF explants after (**C**) two weeks and (**D**) six weeks are illustrated. The white dotted line indicates an accumulation of perilipin positive, red cells. Bar: 100 µm. * *p* < 0.05.

To quantify the number of cells in the vascularized mSVF explants, nuclei were stained with DAPI (Figure 8). No difference in the number of cell nuclei was found. The expression of perilipin, a specific adipocyte marker, was significantly increased in the vascularized mSVF explants after six weeks. While in the mSVF explants after two weeks perilipin only was expressed in very few to almost no cells, mSVF explants after six weeks showed perilipin-positive areas randomly dispersed in the tissue.

### 3.7. FABP4 Regulation

**Figure 9:**
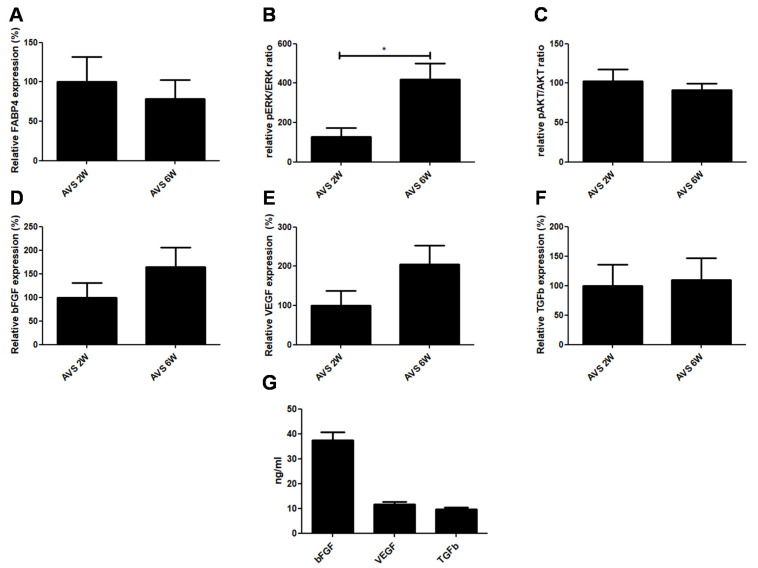
Expression of adipogenesis, proliferation markers and growth factors. (**A**) FABP4 mRNA expression of vascularized mSVF explants after two and six weeks. There was no difference in FABP4 mRNA expression between two and six weeks. (**B**) pERK/ERK and **(C**) pAKT/AKT ratios in vascularized mSVF explants after two and six weeks. While no difference in phosphorylation of Akt was seen, activation of pERK1/2 was significantly increased after six weeks. After two and six weeks, there was no statistical difference in the mRNA expression of (**D**) bFGF, (**E**) VEGF, (**F**) TGFb in vascularized mSVF. (**G**) When cultured, mSVF secreted significant amounts of VEGF, bFGF and TGFb. * *p* < 0.05.

The expression of FABP4 was measured next to evaluate adipogenic regulation of the vascularized mSVF explants after two and six weeks (Figure 9A). Our measurements revealed that FABP4 was expressed in vascularized mSVF explants after two and six weeks with no statistical difference between the two time points.

### 3.8. pAKT/AKT and pERK/ERK

The activation of two major kinases involved in adipose tissue proliferation were investigated. While there was no noticeable difference in the phosphorylation of Akt, there was a significant increase in ERK phosphorylation in the mSVF explanted after six weeks (Figure 9B,C).

### 3.9. Growth Factors

Finally, mRNA and protein levels of common soluble factors VEGF, bFGF and TGFb, which play a key role in the regenerative impact of adipose tissue, were measured. First, mRNA levels were detected in vascularized mSVF explants after two and six weeks. We found that the mRNA of all three growth factors were stably expressed in mSVF after two and six weeks (Figure 9D–F). Although there was no statistical significance, there was a trend towards increased VEGF and bFGF expression after six weeks when compared to two weeks. When fresh mSVF cells were cultured, measurable concentrations of VEGF, bFGF and TGFb were observed (Figure 9G).

## 4. Discussion

For tissue engineering purposes, an AV loop model utilizing a vein/artery graft between the femoral or saphenous vessels is commonly used [16,17]. In the present project, by contrast, we utilized an AV shunt without vein/artery graft interposition to vascularize our mSVF. The feasibility of AV shunts was reported earlier in rabbits [18], rats [18,19] or even mice [20], although mostly for larger femoral vessels and for different approaches. Although the length gain by a graft offers undeniable benefits, earlier studies have shown that the sprouting of new blood vessels primarily originates from the vein and artery in the early stages [17] and later on from the vein, artery and the graft [24]. In our experience, dissecting the saphenous vein and artery in its whole course offers sufficient length to supply the chamber of our dimensions. Admittedly, the length may not be appropriate for larger chambers and higher volumes. Furthermore, we chose relatively large animals (average weight of 380 to 420 g) to maximize the length and size of the vessels. A concern regarding the AV shunting is that increased shear stress, due to the smaller radius of the vascular axis when compared to the AV loop model, may result in higher thrombosis. This, however, was not observed in our studies as thrombosis was only found in one out of thirteen AV shunts.

The great advantage of the AV shunt is the potential reduction in operated rats according to the 3R (replacement, reduction and refinement) principles as no vein graft has to be harvested from the contralateral leg. In theory, two AV shunts can be established in a single rat which would allow comparing two vascularized chambers (e.g., filled with cells on one side and the control on the contralateral side) in one rat diminishing interindividual variation. If only one AV shunt is needed, an intraoperative failed AV shunt (e.g., due to recurrent thrombosis) can be turned into the non-vascularized control group as another AV shunt can be anastomosed on the contralateral leg. This strategy further decreases the potential loss of rats and was used in our study to diminish the number of sacrificed animals. No comparable solution is available in the AV loop model with artery/vein grafts.

We have deliberately not chosen other vascularized control groups in our study as our key interest was to evaluate mSVF proliferation over time and not its superiority to other cell sources or SVF isolation protocols. Beyond that, previous well-conducted studies already showed that minced fat [25] or enzymatically isolated ASCs [26] promoted angiogenesis and tissue growth.

One fundamental issue in the application of fat grafts, SVF cells, and ASCs is the unpredictable absorption rate at the recipient site [27,28]. Fat grafts, SVF cells and ASCs benefit from a scaffold that is able to retain cells, serves as a guide for sprouting capillaries/in-growing cells and assists in the integration of the construct into the recipient site [29,30]. A plethora of scaffolds has been suggested for different clinical applications. In the present study, fibrin hydrogel was used as an mSVF-carrier due to its well-known biocompatibility with ASCs [31], ability to encapsulate cells, rich experience as a scaffold in tissue engineering [32] and wide application in clinical practice [33]. Although fibrin hydrogel is known to be quickly degraded as numerous studies show [34], this downside was less important in our study design as mechanical stability within the first days before onset of cell proliferation/differentiation was considered most important in our study. Similarly, despite the fact that far more innovative hydrogels have been proposed, we selected fibrin hydrogel as a simple but reliable hydrogel as the evaluation of mSVF was our main interest. A concentration of 12.5 mg/mL fibrinogen was chosen as it showed a favorable cell proliferation rate [35] and sufficient hydrogel density [36] when mixed with our mSVF.

In our experiments, mSVF vascularized by AV shunts gained weight over time which indicates that there is an ongoing growth of tissue. Additionally, the histological analysis revealed that vascularized mSVF explants expressed the adipocyte marker perilipin mostly after six weeks, whereas few positive cells were observed after two weeks. Perilipin is a phosphoprotein which is found in the coating of lipid storage droplets of mature adipocytes [37]. Our protocol mainly disrupts mature adipocytes so that the low number of perilipin-positive cells after two weeks may be explained by the fact that precursor cells including ASCs, which do not express perilipin [37], are not fully differentiated to adipocytes at that stage. On the other hand, the perilipin-positive cells did not assume an adipocyte-like cell architecture after six weeks. It appears that six weeks was insufficient for adipocytes to terminally differentiate. The fact that the adipogenesis marker FABP4 was actively expressed after two and six weeks suggests that the mSVF undergoes adipogenesis. Somewhat surprisingly, FABP4 expression, which is commonly regarded a marker for differentiated adipocytes, was not higher in the six-week groups despite the higher perilipin expression. However, earlier studies have reported that FABP4 is also expressed in adipogenic progenitor cells which may have resembled the major share in the mSVF explants after two weeks [38]. Our trichrome staining indicated that there was a significant amount of collagen deposition in the mSVF explants which may be less prominent in collagenase-digested ASCs. The high collagen content was anticipated as mechanical SVF isolation protocols are unable to selectively and equally efficiently disarray adipocytes from the ECM as collagenase digestion [39]. Consequently, the ECM remains in the mSVF fraction and may add to tissue fibrosis, which is an important aspect that needs additional exploration.

To further shed light into kinases of mSVF proliferation, the phosphorylation of ERK1/2 and Akt were analyzed. Akt is a key upstream kinase that governs adipose tissue functions such as lipolysis or glucose uptake [40]. Unlike Akt, ERK1/2 phosphorylation was significantly enhanced after six weeks. The activation of the ERK (a member of the mitogen-activated protein kinase (MAPK) family) pathway is an established route in the proliferation of ASCs [4]. Aside from proliferation, phosphorylated ERK1/2 is believed to contribute to osteogenic differentiation of ASCs [41] and potentially chondrogenic differentiation [42]. Therefore, the ERK pathway may be an interesting target to regulate mSVF differentiation in potential future tissue engineering approaches. Of course, a much more in-depth analysis of pathways may have been possible, but this goes beyond the scope of this translational proof-of-concept study.

It is widely acknowledged that transplanted adipose tissue, SVF and ASCs exert tissue regeneration by the release of cytokines including growth factors [43,44,45]. VEGF, bFGF are well-known soluble factors with strong pro-angiogenic potential which also contribute to adipose tissue growth [46]. The TGF superfamily controls several adipose tissue functions by various pathways. As a negative regulator of adipogenesis, TGFb inhibits differentiation into mature adipocytes but enhances the proliferation of progenitor cells [47]. The stable expression of VEGF, bFGF and TGFb in our construct therefore suggests that growth factor-associated effects may contribute to the proliferation and vascularization of the construct over time. Despite trends towards an increase of bFGF and VEGF expression after six weeks when compared to two weeks, these effects both were statistically non-significant. The most likely explanation is the small sample size plus the high variation when measuring growth factors.

To our knowledge, the only work that has scrutinized mechanically manipulated fat tissue by an arteriovenous loop was recently published by the group around Wayne Morrison [25], who crudely minced and centrifuged adipose tissue instead of disrupting adipocytes. Similar to our observations, Debels et al. also found an increase in weight and perilipin expression of the implanted fat tissue over time. However, in contrast to the aforementioned work, we observed less fat necrosis in our vascularized specimens which may be explained by the markedly smaller amount of tissue used in our experiments which was 0.2 g of mSVF when compared to 1 mL of fat by Debels et al.

## 5. Conclusions

Together, we demonstrate that vascularized mSVF, which is a mixture of SVF cells, small fragments of adipocytes and ECM, proliferates and expresses perilipin mostly after six weeks in vivo. While no difference in FABP4 and Akt phosphorylation was observed between two and six weeks, ERK1/2 activation is significantly enhanced after six weeks. Mechanical SVF also expresses measurable amounts of growth factors that may contribute to adipose tissue proliferation and angiogenesis. Beyond these findings, we can show that the present AV shunt model is a reliable tool for in vivo tissue engineering research that can help to reduce and refine animal experiments.

## Data Availability

Not applicable.

## References

[1] Zuk P.A., Zhu M., Mizuno H., Huang J., Futrell J.W., Katz A.J., Benhaim P., Lorenz H.P., Hedrick M.H. (2001). Multilineage cells from human adipose tissue: Implications for cell-based therapies. Tissue Eng..

[2] Shukla L., Yuan Y., Shayan R., Greening D.W., Karnezis T. (2020). Fat Therapeutics: The Clinical Capacity of Adipose-Derived Stem Cells and Exosomes for Human Disease and Tissue Regeneration. Front. Pharmacol..

[3] Bateman M.E., Strong A.L., Gimble J.M., Bunnell B.A. (2018). Concise Review: Using Fat to Fight Disease: A Systematic Review of Nonhomologous Adipose-Derived Stromal/Stem Cell Therapies. Stem Cells.

[4] Dai R., Wang Z., Samanipour R., Koo K.I., Kim K. (2016). Adipose-Derived Stem Cells for Tissue Engineering and Regenerative Medicine Applications. Stem Cells Int..

[5] Raposio E., Ciliberti R. (2017). Clinical use of adipose-derived stem cells: European legislative issues. Ann. Med. Surg..

[6] Conde-Green A., Kotamarti V.S., Sherman L.S., Keith J.D., Lee E.S., Granick M.S., Rameshwar P. (2016). Shift toward Mechanical Isolation of Adipose-derived Stromal Vascular Fraction: Review of Upcoming Techniques. Plast. Reconstr. Surg. Glob. Open.

[7] Shah F.S., Wu X., Dietrich M., Rood J., Gimble J.M. (2013). A non-enzymatic method for isolating human adipose tissue-derived stromal stem cells. Cytotherapy.

[8] Markarian C.F., Frey G.Z., Silveira M.D., Chem E.M., Milani A.R., Ely P.B., Horn A.P., Nardi N.B., Camassola M. (2014). Isolation of adipose-derived stem cells: A comparison among different methods. Biotechnol. Lett..

[9] Raposio E., Caruana G., Bonomini S., Libondi G. (2014). A novel and effective strategy for the isolation of adipose-derived stem cells: Minimally manipulated adipose-derived stem cells for more rapid and safe stem cell therapy. Plast. Reconstr. Surg..

[10] Copcu H.E. (2022). Indication-based protocols with different solutions for mechanical stromal-cell transfer. Scars Burn. Heal..

[11] Tonnard P., Verpaele A., Peeters G., Hamdi M., Cornelissen M., Declercq H. (2013). Nanofat grafting: Basic research and clinical applications. Plast. Reconstr. Surg..

[12] Trivisonno A., Alexander R.W., Baldari S., Cohen S.R., Di Rocco G., Gentile P., Magalon G., Magalon J., Miller R.B., Womack H. (2019). Intraoperative Strategies for Minimal Manipulation of Autologous Adipose Tissue for Cell- and Tissue-Based Therapies: Concise Review. Stem Cells Transl. Med..

[13] Pallua N., Grasys J., Kim B.S. (2018). Enhancement of Progenitor Cells by Two-Step Centrifugation of Emulsified Lipoaspirates. Plast. Reconstr. Surg..

[14] Pallua N., Kim B.S. (2020). Microfat and Lipoconcentrate for the Treatment of Facial Scars. Clin. Plast. Surg..

[15] Lujan-Hernandez J., Appasani R., Sullivan K., Siegel-Reamer L., Lalikos J.F. (2017). Experimental In-Vivo Models Used in Fat Grafting Research for Volume Augmentation in Soft Tissue Reconstruction. Arch. Plast. Surg..

[16] Weigand A., Horch R.E., Boos A.M., Beier J.P., Arkudas A. (2018). The Arteriovenous Loop: Engineering of Axially Vascularized Tissue. Eur. Surg. Res. Eur. Chir. Forsch. Rech. Chir. Eur..

[17] Lokmic Z., Stillaert F., Morrison W.A., Thompson E.W., Mitchell G.M. (2007). An arteriovenous loop in a protected space generates a permanent, highly vascular, tissue-engineered construct. FASEB J. Off. Publ. Fed. Am. Soc. Exp. Biol..

[18] Spater T., Ampofo E., Menger M.D., Laschke M.W. (2020). Combining Vascularization Strategies in Tissue Engineering: The Faster Road to Success?. Front. Bioeng. Biotechnol..

[19] Croatt A.J., Grande J.P., Hernandez M.C., Ackerman A.W., Katusic Z.S., Nath K.A. (2010). Characterization of a model of an arteriovenous fistula in the rat: The effect of L-NAME. Am. J. Pathol..

[20] Wong R., Donno R., Leon-Valdivieso C.Y., Roostalu U., Derby B., Tirelli N., Wong J.K. (2019). Angiogenesis and tissue formation driven by an arteriovenous loop in the mouse. Sci. Rep..

[21] Kirk R.G.W. (2018). Recovering The Principles of Humane Experimental Technique: The 3Rs and the Human Essence of Animal Research. Sci. Technol. Hum. Values.

[22] Kao H.K., Hsu H.H., Chuang W.Y., Chang K.P., Chen B., Guo L. (2015). Experimental study of fat grafting under negative pressure for wounds with exposed bone. Br. J. Surg..

[23] Taylor S.C., Nadeau K., Abbasi M., Lachance C., Nguyen M., Fenrich J. (2019). The Ultimate qPCR Experiment: Producing Publication Quality, Reproducible Data the First Time. Trends Biotechnol..

[24] Kneser U., Polykandriotis E., Ohnolz J., Heidner K., Grabinger L., Euler S., Amann K.U., Hess A., Brune K., Greil P. (2006). Engineering of vascularized transplantable bone tissues: Induction of axial vascularization in an osteoconductive matrix using an arteriovenous loop. Tissue Eng..

[25] Debels H., Palmer J., Han X.L., Poon C., Abberton K., Morrison W. (2020). In vivo tissue engineering of an adipose tissue flap using fat grafts and Adipogel. J. Tissue Eng. Regen. Med..

[26] Matsuda K., Falkenberg K.J., Woods A.A., Choi Y.S., Morrison W.A., Dilley R.J. (2013). Adipose-derived stem cells promote angiogenesis and tissue formation for in vivo tissue engineering. Tissue Eng. Part A.

[27] Chung M.T., Hyun J.S., Lo D.D., Montoro D.T., Hasegawa M., Levi B., Januszyk M., Longaker M.T., Wan D.C. (2013). Micro-computed tomography evaluation of human fat grafts in nude mice. Tissue Eng. Part C Methods.

[28] Gonzalez A.M., Lobocki C., Kelly C.P., Jackson I.T. (2007). An alternative method for harvest and processing fat grafts: An in vitro study of cell viability and survival. Plast. Reconstr. Surg..

[29] Mahoney C.M., Imbarlina C., Yates C.C., Marra K.G. (2018). Current Therapeutic Strategies for Adipose Tissue Defects/Repair Using Engineered Biomaterials and Biomolecule Formulations. Front. Pharmacol..

[30] Dryden G.W., Boland E., Yajnik V., Williams S. (2017). Comparison of Stromal Vascular Fraction with or Without a Novel Bioscaffold to Fibrin Glue in a Porcine Model of Mechanically Induced Anorectal Fistula. Inflamm. Bowel Dis..

[31] Chung E., Rytlewski J.A., Merchant A.G., Dhada K.S., Lewis E.W., Suggs L.J. (2015). Fibrin-based 3D matrices induce angiogenic behavior of adipose-derived stem cells. Acta Biomater..

[32] Bensaid W., Triffitt J.T., Blanchat C., Oudina K., Sedel L., Petite H. (2003). A biodegradable fibrin scaffold for mesenchymal stem cell transplantation. Biomaterials.

[33] Huang S., Fu X. (2010). Naturally derived materials-based cell and drug delivery systems in skin regeneration. J. Control. Release Off. J. Control. Release Soc..

[34] Rowe S.L., Lee S., Stegemann J.P. (2007). Influence of thrombin concentration on the mechanical and morphological properties of cell-seeded fibrin hydrogels. Acta Biomater..

[35] Mooney R., Tawil B., Mahoney M. (2010). Specific fibrinogen and thrombin concentrations promote neuronal rather than glial growth when primary neural cells are seeded within plasma-derived fibrin gels. Tissue Eng. Part A.

[36] Brown A.C., Barker T.H. (2014). Fibrin-based biomaterials: Modulation of macroscopic properties through rational design at the molecular level. Acta Biomater..

[37] Greenberg A.S., Egan J.J., Wek S.A., Garty N.B., Blanchette-Mackie E.J., Londos C. (1991). Perilipin, a major hormonally regulated adipocyte-specific phosphoprotein associated with the periphery of lipid storage droplets. J. Biol. Chem..

[38] Shan T., Liu W., Kuang S. (2013). Fatty acid binding protein 4 expression marks a population of adipocyte progenitors in white and brown adipose tissues. FASEB J. Off. Publ. Fed. Am. Soc. Exp. Biol..

[39] Sun M., He Y., Zhou T., Zhang P., Gao J., Lu F. (2017). Adipose Extracellular Matrix/Stromal Vascular Fraction Gel Secretes Angiogenic Factors and Enhances Skin Wound Healing in a Murine Model. BioMed Res. Int..

[40] Siddle K. (2011). Signalling by insulin and IGF receptors: Supporting acts and new players. J. Mol. Endocrinol..

[41] Liu Q., Cen L., Zhou H., Yin S., Liu G., Liu W., Cao Y., Cui L. (2009). The role of the extracellular signal-related kinase signaling pathway in osteogenic differentiation of human adipose-derived stem cells and in adipogenic transition initiated by dexamethasone. Tissue Eng. Part A.

[42] Kim B.S., Kang K.S., Kang S.K. (2010). Soluble factors from ASCs effectively direct control of chondrogenic fate. Cell Prolif..

[43] Kim B.S., Gaul C., Paul N.E., Dewor M., Stromps J.P., Hwang S.S., Nourbakhsh M., Bernhagen J., Rennekampff H.O., Pallua N. (2016). The Effect of Lipoaspirates on Human Keratinocytes. Aesthetic Surg. J..

[44] Pallua N., Pulsfort A.K., Suschek C., Wolter T.P. (2009). Content of the growth factors bFGF, IGF-1, VEGF, and PDGF-BB in freshly harvested lipoaspirate after centrifugation and incubation. Plast. Reconstr. Surg..

[45] Pallua N., Serin M., Wolter T.P. (2014). Characterisation of angiogenetic growth factor production in adipose tissue-derived mesenchymal cells. J. Plast. Surg. Hand Surg..

[46] Rophael J.A., Craft R.O., Palmer J.A., Hussey A.J., Thomas G.P., Morrison W.A., Penington A.J., Mitchell G.M. (2007). Angiogenic growth factor synergism in a murine tissue engineering model of angiogenesis and adipogenesis. Am. J. Pathol..

[47] Zamani N., Brown C.W. (2011). Emerging roles for the transforming growth factor-{beta} superfamily in regulating adiposity and energy expenditure. Endocr. Rev..

